# Supplementary material for: Multi‐dataset identification of innovative feature genes and molecular mechanisms in keratoconus
Source: J Cell Mol Med. 2024 Sep 19;28(18):e70079. doi: 10.1111/jcmm.70079 (PMC11412914; doi:10.1111/jcmm.70079)
Supplement: Supplementary file 2 — Material S2. [file JCMM-28-e70079-s002.zip › 7.GSEA analysis/5.RNF217-AS1/KEGG_BUTANOATE_METABOLISM.html]

Details for gene set KEGG\_BUTANOATE\_METABOLISM[GSEA]

|  || Dataset | RNF217-AS1.group.cls#h\_versus\_l |
| Phenotype | group.cls#h\_versus\_l |
| Upregulated in class | l |
| GeneSet | KEGG\_BUTANOATE\_METABOLISM |
| Enrichment Score (ES) | -0.46928972 |
| Normalized Enrichment Score (NES) | -1.3715996 |
| Nominal p-value | 0.12981744 |
| FDR q-value | 0.35464853 |
| FWER p-Value | 0.896 |
Table: GSEA Results Summary

  

Fig 1: Enrichment plot: KEGG\_BUTANOATE\_METABOLISM      
 Profile of the Running ES Score & Positions of GeneSet Members on the Rank Ordered List

  

| SYMBOL | TITLE | RANK IN GENE LIST | RANK METRIC SCORE | RUNNING ES | CORE ENRICHMENT || 1 | ACSM1 | na | 3404 | 0.275 | -0.0402 | No |
| 2 | ACSM2A | na | 7111 | 0.150 | -0.1154 | No |
| 3 | ABAT | na | 7227 | 0.146 | -0.0879 | No |
| 4 | ACSM4 | na | 7575 | 0.138 | -0.0687 | No |
| 5 | PDHA1 | na | 8282 | 0.121 | -0.0635 | No |
| 6 | HMGCS2 | na | 9330 | 0.099 | -0.0729 | No |
| 7 | HMGCL | na | 10116 | 0.085 | -0.0777 | No |
| 8 | PDHA2 | na | 12139 | 0.053 | -0.1247 | No |
| 9 | BDH1 | na | 14766 | 0.022 | -0.1958 | No |
| 10 | ACSM5 | na | 15966 | 0.009 | -0.2285 | No |
| 11 | L2HGDH | na | 16237 | 0.006 | -0.2349 | No |
| 12 | ACAT2 | na | 17362 | -0.005 | -0.2662 | No |
| 13 | AACS | na | 17765 | -0.009 | -0.2760 | No |
| 14 | HMGCS1 | na | 19419 | -0.024 | -0.3185 | No |
| 15 | PDHB | na | 19823 | -0.028 | -0.3241 | No |
| 16 | ALDH3A2 | na | 20121 | -0.031 | -0.3261 | No |
| 17 | HADHA | na | 20646 | -0.036 | -0.3336 | No |
| 18 | ECHS1 | na | 20997 | -0.040 | -0.3352 | No |
| 19 | GAD2 | na | 21907 | -0.050 | -0.3510 | No |
| 20 | ACADS | na | 22353 | -0.054 | -0.3523 | No |
| 21 | ACAT1 | na | 22576 | -0.057 | -0.3467 | No |
| 22 | OXCT1 | na | 22630 | -0.058 | -0.3361 | No |
| 23 | ALDH9A1 | na | 23254 | -0.065 | -0.3404 | No |
| 24 | ALDH7A1 | na | 25259 | -0.095 | -0.3782 | No |
| 25 | HADH | na | 26099 | -0.109 | -0.3794 | No |
| 26 | EHHADH | na | 27336 | -0.133 | -0.3870 | No |
| 27 | ALDH1B1 | na | 28642 | -0.166 | -0.3896 | No |
| 28 | OXCT2 | na | 31408 | -0.259 | -0.4147 | Yes |
| 29 | BDH2 | na | 31580 | -0.267 | -0.3633 | Yes |
| 30 | ALDH5A1 | na | 32732 | -0.340 | -0.3248 | Yes |
| 31 | GAD1 | na | 33122 | -0.370 | -0.2581 | Yes |
| 32 | ACSM3 | na | 33224 | -0.378 | -0.1813 | Yes |
| 33 | ALDH2 | na | 33631 | -0.423 | -0.1038 | Yes |
| 34 | AKR1B10 | na | 34485 | -0.642 | 0.0070 | Yes |
Table: GSEA details [plain text format]

  

Fig 2: KEGG\_BUTANOATE\_METABOLISM      
 Blue-Pink O' Gram in the Space of the Analyzed GeneSet

  

Fig 3: KEGG\_BUTANOATE\_METABOLISM: Random ES distribution      
 Gene set null distribution of ES for **KEGG\_BUTANOATE\_METABOLISM**
